# Common inversion polymorphism at 17q21.31 affects expression of multiple genes in tissue-specific manner

**DOI:** 10.1186/1471-2164-13-458

**Published:** 2012-09-06

**Authors:** Simone de Jong, Iouri Chepelev, Esther Janson, Eric Strengman, Leonard H van den Berg, Jan H Veldink, Roel A Ophoff

**Affiliations:** 1 Department of Medical Genetics, University Medical Center Utrecht, Utrecht, 3584 CG, The Netherlands; 2 Department of Psychiatry, Rudolf Magnus Institute of Neuroscience, University Medical Center Utrecht, Utrecht, 3508 GA, The Netherlands; 3 Center for Neurobehavioral Genetics, University of California Los Angeles, Box 951761, Gonda #4357C 695 Charles E. Young Drive South, Los Angeles, CA, 90095, USA; 4 Laboratory of Molecular Immunology, National Heart, Lung and Blood Institute, National Institute of Health, Bethesda, MD, 20892, USA; 5 Department of Neurology, Rudolf Magnus Institute of Neuroscience, University Medical Center Utrecht, Utrecht, 3584 CX, The Netherlands

## Abstract

**Background:**

Chromosome 17q21.31 contains a common inversion polymorphism of approximately 900 kb in populations with European ancestry. Two divergent *MAPT* haplotypes, H1 and H2 are described with distinct linkage disequilibrium patterns across the region reflecting the inversion status at this locus. The *MAPT* H1 haplotype has been associated with progressive supranuclear palsy, corticobasal degeneration, Parkinson’s disease and Alzheimer’s disease, while the H2 is linked to recurrent deletion events associated with the 17q21.31 microdeletion syndrome, a disease characterized by developmental delay and learning disability.

**Results:**

In this study, we investigate the effect of the inversion on the expression of genes in the 17q21.31 region. We find the expression of several genes in and at the borders of the inversion to be affected; specific either to whole blood or different regions of the human brain. The H1 haplotype was found to be associated with an increased expression of *LRRC37A4*, *PLEKH1M* and *MAPT*. In contrast, a decreased expression of *MGC57346*, *LRRC37A* and *CRHR1* was associated with H1.

**Conclusions:**

Studies thus far have focused on the expression of *MAPT* in the inversion region. However, our results show that the inversion status affects expression of other genes in the 17q21.31 region as well. Given the link between the inversion status and different neurological diseases, these genes may also be involved in disease pathology, possibly in a tissue-specific manner.

## Background

**Figure 1:**
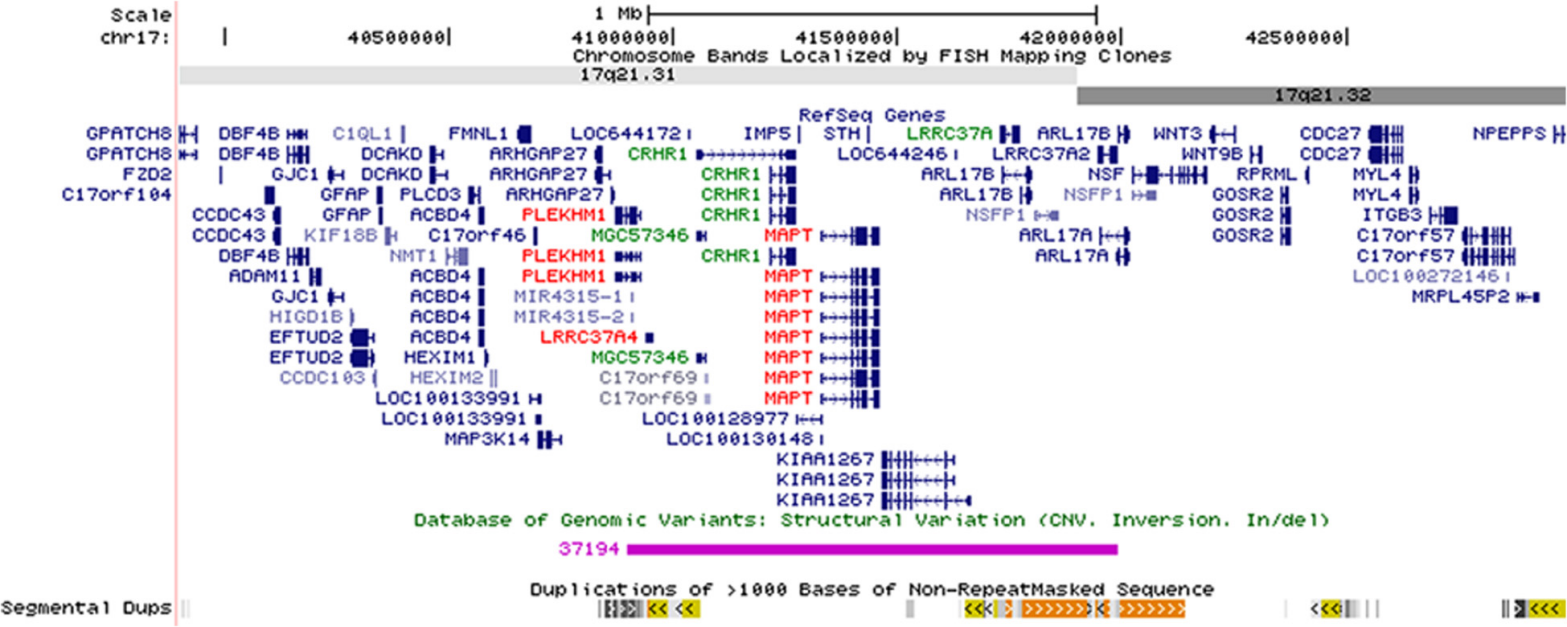
**17q21.31 inversion region.** Schematic overview of the 17q21.31 region in the direction orientation corresponding with the H1 haplotype and the *cis*-region of 1 MB each side (Chr17:39,899,921-42,989,253; UCSC browser, March 2006 assembly, http://genome.ucsc.edu). Pink segment shows the 40850001–41850000 region. Expression of genes colored red is increased in the H1/H1 haplotype (*LRRC37A4* in blood, *PLEKHM1* and *MAPT* in one or more brain regions), those in green decreased (*MGC57346* in blood, *CRHR1* and *LRRC37A* in one or more brain regions).

The chromosomal band 17q21.31 contains a common inversion polymorphism linked with neurodegenerative diseases including progressive supranuclear palsy [1], corticobasal degeneration [2], Parkinson’s disease [3], and Alzheimer’s disease [4]. This inversion of approximately 900 kb is mostly present in populations with European ancestry (Figure 1) [5,6]. This region contains a number of genes, including corticotrophin releasing hormone receptor 1 (*CRHR1*) and microtubule-associated protein tau (*MAPT*). Two divergent *MAPT* haplotypes, H1 and H2 are described with distinct linkage disequilibrium patterns across the region reflecting the inversion status at this locus. The H2 haplotype is inverted and is relatively common in Europeans (~20%), however, almost absent in African and Asian populations. This configuration is associated with fecundity and appears to be under positive selection in European populations [6].

Specific H1 haplotypes are associated with the neurodegenerative disorders, whereas the H2 haplotype is linked to recurrent deletion events resulting in the 17q21.31 microdeletion syndrome, characterized by developmental delay and learning disability [7,8]. The H1 haplotype is linked with an increased expression of *MAPT*, resulting in overproduction and aggregation of hyperphosphorylated protein Tau in neuronal cell bodies, which is linked to disease pathology of a number of neurodegenerative disorders [9-12]. However, little is known about the regulation of expression levels of other genes in the region.

In this study we used single nucleotide polymorphism (SNP) genotype data to reconstruct inversion haplotypes in the chromosome 17q21.31 region and studied the effect of the inversion status on gene expression of all known genes in the region in whole blood and different regions of the human brain.

## Results

### Regulation of expression in whole blood

**Figure 2:**
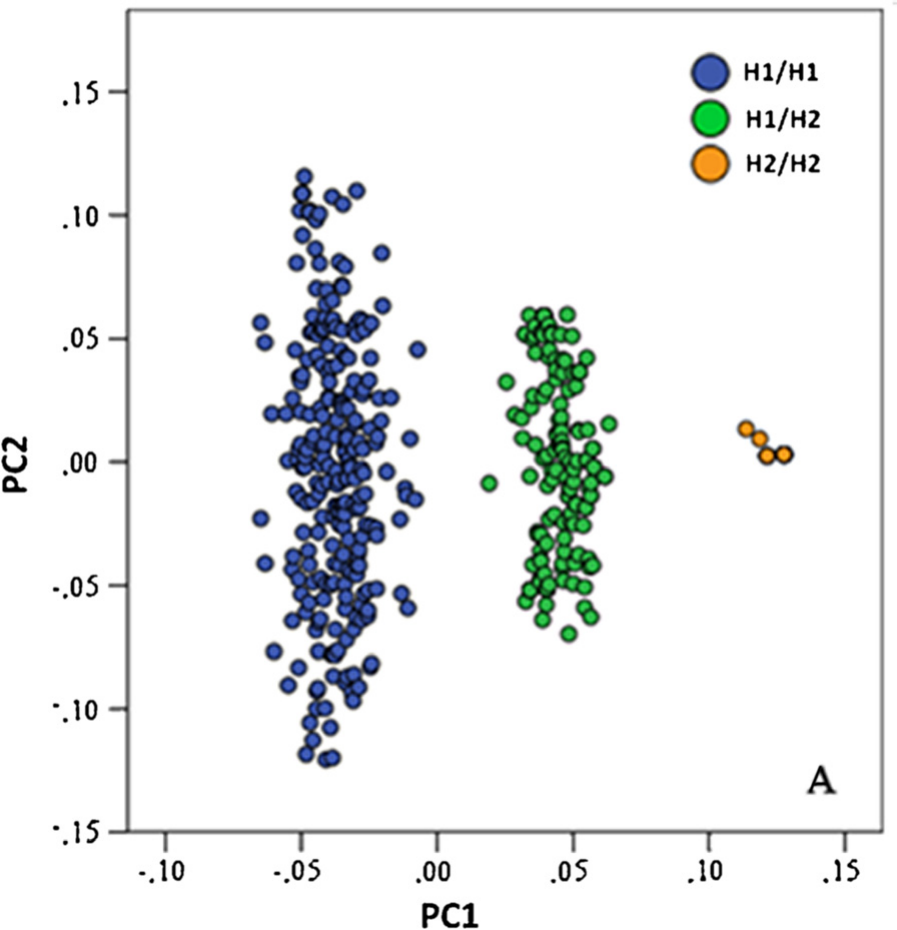
**The 17q21.31 inversion haplotypes in the whole blood dataset.** Scatterplot of PC1 and PC2 values in the 17q21.31 region (constructed using 38 SNPs in the 40,850,001-41,850,000 interval). Individuals fall into three clusters, representing H1/H1 (blue), H1/H2 (green) and H2/H2 (orange).

A principal component analysis (PCA) was applied to 38 SNPs in the 40,850,001-41,850,000 region on chromosome 17. The first principal component (PC1) represents the 17q21.31 inversion genotypes homozygous H1, heterozygous H1/H2 and homozygous H2/H2. Of the 437 individuals, 6 were excluded because of ambiguity in master genotype call (>3SD from mean PC1 value of genotype cluster). This resulted in three distinct clusters of individuals, representing H1/H1 (252 individuals, 59%), H1/H2 (160 individuals, 37%) and H2/H2 (19 individuals, 4%) genotypes (Hardy Weinberg *p* = 0.31), depicted in Figure 2. For 22 of the 56 genes in the region, 28 gene expression probes were available in the blood dataset (full list in Additional file 1: Table S1).

**Figure 3:**
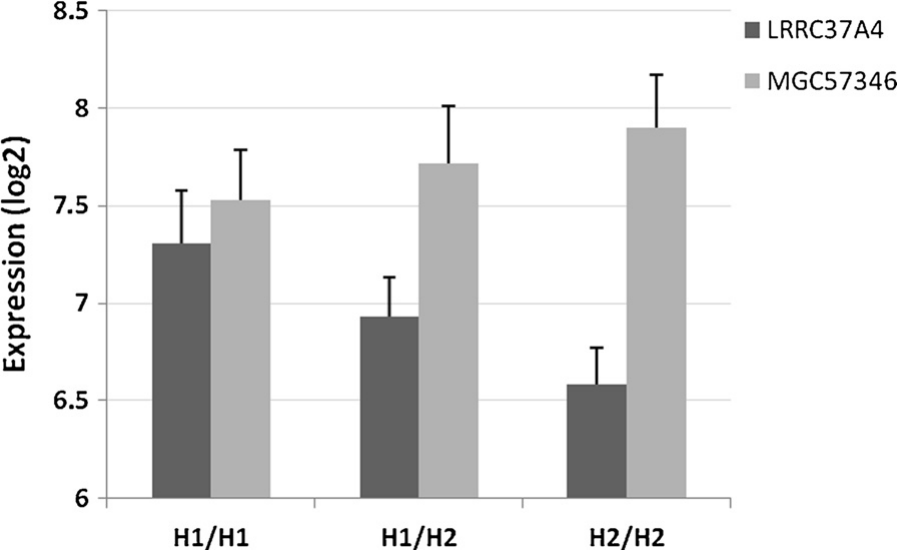
**Expression changes associated with the 17q21.31 inversion haplotypes in the whole blood dataset.** Barplot shows the mean log2 expression values (error bars represent standard deviation) of *LRRC37A4* and *MGC57346* for H1/H1, H1/H2 and H2/H2 genotypes.

A linear regression analysis showed a positive association of *LRRC37A4* (B = 0.37, *p* = 1.4 × 10^-55^, Figure 3) with the number of H1 alleles. In contrast, *MGC57346* was negatively associated with the H1/H1 genotype (B = −0.19, *p* = 1.2 × 10^-16^, Figure 3) Results are given in Table 1. We did not detect expression of the *MAPT* gene in blood.

**Table 1:** Expression probes associated with 17q21.31 inversion region in different datasets

		**Whole blood**		
	Probe ID	t	B (95% CI)	p
**LRRC37A4**	ILMN_2393693	18.29	0.37 (0.33, 0.41)	1.4 x 10^-55^
**MGC57346**	ILMN_1784428	−8.63	−0.19 (−0.24, -0.15)	1.2 x 10^-16^
**PLEKHM1**	ILMN_1709549	ns	ns	ns
**MAPT**	dt	dt	dt	dt
**LRRC37A**	ILMN_1783673	ns	ns	ns
**CRHR1**	ILMN_1732426	ns	ns	ns
		**Cerebellum**		
	Probe ID	t	B (95% CI)	p
**LRRC37A4**	n/a	n/a	n/a	n/a
**MGC57346**	n/a	n/a	n/a	n/a
**PLEKHM1**	ILMN_1709549	4.98	0.18 (0.11, 0.25)	1.9 x 10^-6^
**MAPT**	ILMN_1710903	6.01	0.36 (0.24, 0.48)	1.6 x 10^-8^
**LRRC37A**	ILMN_1783673	ns	ns	ns
**CRHR1**	ILMN_1753706	−4.18	−0.09 (−0.13, -0.05)	5.2 x 10^-5^
		**Frontal Cortex**		
	Probe ID	t	B (95% CI)	p
**LRRC37A4**	n/a	n/a	n/a	n/a
**MGC57346**	n/a	n/a	n/a	n/a
**PLEKHM1**	ILMN_1709549	ns	ns	ns
**MAPT**	ILMN_1710903	4.76	0.34 (0.20, 0.48)	4.8 x 10^-6^
**LRRC37A**	ILMN_1783673	−7.92	−0.25 (−0.32, -0.19)	7.4 x 10^-13^
**CRHR1**	ILMN_1753706	dt	dt	dt
		**Temporal Cortex**		
	Probe ID	t	B (95% CI)	p
**LRRC37A4**	n/a	n/a	n/a	n/a
**MGC57346**	n/a	n/a	n/a	n/a
**PLEKHM1**	ILMN_1709549	n/a	ns	ns
**MAPT**	ILMN_1710903	ns	ns	ns
**LRRC37A**	ILMN_1783673	−9.15	−0.29 (−0.35, -0.23)	6.9 x 10^-16^
**CRHR1**	ILMN_1753706	dt	dt	dt
		**Pons**		
	Probe ID	t	B (95% CI)	p
**LRRC37A4**	n/a	n/a	n/a	n/a
**MGC57346**	n/a	n/a	n/a	n/a
**PLEKHM1**	ILMN_1709549	ns	ns	ns
**MAPT**	ILMN_1710903	ns	ns	ns
**LRRC37A**	ILMN_1783673	−6.03	−0.17 (−0.23, -0.12)	1.5 x 10^-8^
**CRHR1**	ILMN_1753706	dt	dt	dt

The genes found to be associated to the 17q21.31 inversion haplotypes are listed in the first column with their corresponding probe IDs (Illumina H12.v3 in blood and Illumina H8.v2 in brain). The genes have been tested in two datasets and results of linear regression are given, t, B (with 95% CI) and *p*-values. The B values indicate the actual change in expression associated with each copy of the H1 allele (0, 1 or 2). The corresponding unadjusted *p*-values are listed when significant. Not all genes were available or detected in each dataset; n/a = not available on array, dt = did not meet detection threshold in tissue, ns = not significant. All probes for the genes in the 17q21.31 region are listed in Additional file 1: Table S1.

### Regulation of expression in human brain

We consulted a publically available human brain expression dataset consisting of frontal cortex, temporal cortex, cerebellum and pons of 144 individuals [13]. Master genotypes of the chromosome 17q21.31 inversion were reconstructed using PC1 values of 60 SNPs in the 40,850,001-41,850,000 interval. This resulted in three distinct clusters of individuals, representing H1/H1 (77 individuals, 53%), H1/H2 (54 individuals, 38%) and H2/H2 (13 individuals, 9%) genotypes (Hardy Weinberg *p* = 0.43).

For 36 of the 56 genes in the inversion region, 40 expression probes were available in every brain area (full list in Additional file 1: Table S1). A linear regression analysis of allele dosage was performed for each brain area separately (Table 1). In line with literature, we found a higher expression of *MAPT* to be associated with the H1/H1 genotype in frontal cortex (B = 0.34, *p* = 4.8 × 10^-6^) and cerebellum (B = 0.36, *p* = 1.6 × 10^-8^). In addition, the H1/H1 genotype was also associated with increased expression of *PLEKHM1* in cerebellum (B = 0.18, *p* = 1.9 × 10^-6^). In contrast, lower expression of *CRHR1* was associated with this genotype in cerebellum (B = −0.89, *p* = 5.2 × 10^-5^), while undetected in the other brain regions. Finally, decreased expression of *LRRC37A* was found to be associated with the H1/H1 genotype in frontal cortex (B = −0.25, *p* = 7.4 × 10^-13^), temporal cortex (B = −0.29, *p* = 6.9 × 10^-16^) and pons (B = −0.17, *p* = 1.5 × 10^-8^).

No polymorphic SNPs were detected in the probe sequence that could have confounded the hybridization signal. When aligning the *LRRC37A* probe sequence (50 nucleotides, identical on H12.v3 and H8.v2 Illumina beadarrays) to refseq RNA sequences (NCBI BLAST; http://blast.ncbi.nlm.nih.gov/Blast.cgi), it was found that this probe aligns significantly with not only *LRRC37A* (100%), but also *LRRC37A2* (100%), *LRRC37A3* (100%) and *LRRC37A4* (94%). Therefore, the strong association can be the result of non-specific binding to more than one target gene in this gene family. Alignment of all other significant probes sequences, including *LRRC37A4*, did not suggest non-specific binding.

## Discussion

The chromosome 17q21.31 inversion of the *MAPT* (microtubule-associated protein Tau) locus represents one of the most structurally complex and evolutionarily dynamic regions of the genome [14]. The distinct clades of haplotypes (H1 and H2) represent the direct and inverted orientation of the inversion, each with different functional impacts. Specific H1 haplotypes are associated with neurodegenerative disorders such as progressive supranuclear palsy [1] and Parkinson’s disease [3], whereas the H2 haplotype is associated with recurrent microdeletions resulting in the 17q21.31 microdeletion syndrome [7,8]. Neurodegenerative diseases associated with the H1 haplotype exhibit aggregation of hyperphosphorylated protein Tau in neuronal cell bodies [2,12].

Gene expression differences have been described for *MAPT,* but there has been no systematic approach to study the effect of inversion status on expression of the other genes at this locus. We used principal component analysis to identify inversion haplotypes at chromosome 17q21.31, and observed that the effect of inversion status is not limited to *MAPT* expression levels, but also affects several other genes in the 17q21.31 region. In line with literature, we found increased expression of *MAPT* to be associated with the number of H1 alleles in brain. However, we only observed this in frontal cortex and cerebellum, suggesting that regulation of this gene may differ between brain regions. A previous study identified a specific sequence variant in *MAPT* (htSNP167/rs242557) in the 17q21.31 region on the H1 haplotype regulating the expression of *MAPT* in neuronal and non-neuronal cell lines [11]. In this study we focused on the effect of the entire inversion on gene expression with use of a robust principal component analysis strategy. Genotype data of this particular SNP was available in the brain dataset but was not significantly associated with gene expression values.

Importantly, we observed that genes other than *MAPT* are functionally regulated by the inversion haplotypes as well and may therefore be of importance in diseases associated with the inversion region.

The expression of *CRHR1* (corticotrophin releasing hormone receptor 1) is significantly decreased in the H1/H1 haplotype and that of *PLEKHM1* (pleckstrin homology domain containing, family M (with RUN domain) member 1) increased. These associations were found in cerebellum only. The *PLEKHM1* gene is involved in osteopertrosis by affecting vesicular transport and therefore osteoclast-osteoblast cross-talk [15]. Currently there is no functional data available on *MGC57346* (hypothetical protein LOC401884) that was found to be differentially expressed due to inversion status in whole blood. *MGC57346* consists of 4 exons of which 3 are shared with the long isoform of *CRHR1* (Figure 1). It is therefore possible that these are different splice forms of a single gene, which would suggest that the concurrent association findings with inversion status represent a single event. The fact that directionality of *MGC57346* expression in blood and *CRHR1* expression in cerebellum is the same in this study supports this view. The *CRHR* gene is a critical part of the hypothalamic-pituitary-adrenal (HPA) axis that mediates stress response and has been implicated in the pathophysiology of stress-related psychiatric disorders. Of the two receptors in this system (*CRHR1* and *CRHR2*), overactivity of *CRHR1* in anxiety and depression has been a consistent finding in animal studies [16]. In human, there is evidence for an interaction of *CRHR1* function and stressful life events on vulnerability to depression and alcoholism through regulation of HPA-axis and possibly additional interaction with serotonin transporter loci [17]. In addition, multiple sclerosis (MS) has been associated to HPA-axis activity, specifically genetic variants in *CRHR1*[18]. We find increased expression of *CRHR1* associated with H2 configuration, suggesting that *CRHR1* activity and/or stress response might also be altered in or contributing to the H2 related phenotypes such as developmental delay and learning disability.

There is no functional data available for leucine rich repeat containing 37, member A4 (*LRRC37A4*) expressed in whole blood and *LRRC37A* (leucine rich repeat containing 37A) expressed in brain. For both genes we observed a significant association with inversion status, however, with opposite effects. It is important to note that the *LRRC37* gene family is located at either inversion breakpoints and is therefore likely to be affected by copy number variation that are associated with 17q21.31 inversion status [14] (Figure 1). Of the LRRC37 family, member A4 (*LRRC37A4*) in particular has been shown to be most variable in copy number [19]. For these reasons, the association between inversion status and gene expression levels of these genes could be entirely due to differences in copy number linked to H1 and H2 haplotypes.

A recent study finds a strong association between germline hypomethylation and genomic instability, describing that DNA methylation deserts are highly enriched for structural rearrangements [20]. The authors report that rare CNVs that are associated with several neuropsychiatric disorders are significantly linked with local hypomethylation. In fact, germline hypomethylation seems to play a more important role in chromosomal rearrangement than the presence of segmental duplications. Future studies should address whether inversion status of the 17q21.31 region can be linked to (large-scale) changes in epigenetic tags.

## Conclusions

In conclusion, our results indicate that the chromosome 17q21.31 inversion polymorphism associated with several neurodegenerative disorders affects the expression of multiple genes besides *MAPT* in a tissue-specific manner. It is therefore likely that these other genes may also play a role in pathophysiology of these neurodegenerative disorders.

## Methods

### Whole blood dataset

The whole blood dataset consisting of 437 healthy controls is described elsewhere [21,22]. In short, this data set consists of 244 males and 193 females with a mean age of 62 years, who where recruited as controls in a genetic study of amyotrophic lateral sclerosis. These control subjects were selected for being in good general health and unaffected with neurological and neurodegenerative diseases. Genotypes were generated on the Illumina 370 k chip according to manufacturers’ protocol at deCODE genetics in Iceland. QC included missing genotypes per individual <0.05, genotype rate per SNP >0.05, MAF >0.05, Hardy Weinberg *p*<0.00001. Gene expression data for this control dataset was generated using the Illumina H-12.v3 beadarray and quantile normalized and log2 transformed using the PreprocessCore package in R [23]. Expression probes were filtered for a mean detection value > 0.90.

### Brain datasets

We consulted a publically available brain expression dataset of 144 individuals [13]. Gene expression data was generated on Illumina H-8.v2 beadarrays (GEO; GSE15745) and genotype data on Illumina 550 k chips (dbGAP; phs000249.v1.p1). Data are available for four different brain regions; cerebellum, frontal cortex, temporal cortex and pons. Data was log2 transformed and quantile normalized using the Lumi package for R [24]. Expression data was filtered for each brain region separately with a detection *p*-value threshold of 0.01.

### Principal component analysis

We applied a principal component analysis (PCA) using SNP data to reconstruct the inversion genotypes homozygous H1, heterozygous H1/H2, and homozygous H2. The first two principal components (PC1 and PC2) were calculated with genotypes numerically encoded as m/m = 0, m/M = 1, M/M = 2, where m and M are minor and major alleles, respectively, in the 17q21.31 region; 40,850,001-41,850,000 (corresponding to coordinates in UCSC assembly March 2006; http://genome.ucsc.edu) [8]. We centered and normalized the genotype matrix and used it for PCA as described by Price et al. [25].

### Linear regression

A linear regression analysis of allele dosage (H1/H1 = 2, H1/H2 = 1 and H2/H2 = 0) was performed on transcripts in the 17q21.31 *cis*-region; defined as 1 MB on either side of the inversion (39,899,921-42,989,253, Figure 1), containing 56 genes (Refseq genes from UCSC browser, March 2006 assembly; http://genome.ucsc.edu) listed in Additional file 1: Table S1. In the whole blood dataset, age and gender were taken covariates. In brain, covariates also included post-mortem interval, brain bank and hybridization batch. We report the unadjusted *p*-value and B value, indicating the actual change in expression associated with each copy of the H1 allele (0, 1 or 2). Bonferroni correction was applied to determine significance thresholds for the number of probes tested; *p* < 0.05/28 = 0.0018 for the whole blood dataset and *p* < 0.05/40 = 0.0017 for each brain region, assuming independence between regions.

Significant expression probes were subsequently tested for common polymorphic SNPs in the 50-mer probe sequence based on genomic location (provided by Illumina) and Hapmap SNPs release 27. The threshold for common SNPs is minor allele frequency (MAF) >1%.

## Competing interests

The authors declare that they have no competing interests.

## Authors’ contributions

SDJ performed statistical analyses and drafted the manuscript. IC generated inversion haplotypes. EJ and ES provided technical support. LHvdB and JHV prepared datasets for analysis. RAO conceived of the study, participated in its design and helped to draft the manuscript. All authors read and approved the final manuscript.

## Supplementary Material

Additional file 1 **Table S1.** This table contains a list of genes in the 17q21.31 region and the expression probes for these genes in both datasets. ProbeIDs colored red are expressed above detection level in the corresponding dataset. Black probes represent probes available on the array, but not detectable in the tissue studied. Click here for file
